# The Recently Identified P2Y-Like Receptor GPR17 Is a Sensor of Brain Damage and a New Target for Brain Repair

**DOI:** 10.1371/journal.pone.0003579

**Published:** 2008-10-31

**Authors:** Davide Lecca, Maria Letizia Trincavelli, Paolo Gelosa, Luigi Sironi, Paolo Ciana, Marta Fumagalli, Giovanni Villa, Claudia Verderio, Carlotta Grumelli, Uliano Guerrini, Elena Tremoli, Patrizia Rosa, Serena Cuboni, Claudia Martini, Annalisa Buffo, Mauro Cimino, Maria P. Abbracchio

**Affiliations:** 1 Department of Pharmacological Sciences, University of Milan, Milan, Italy; 2 Department of Psychiatry, Neurobiology, Pharmacology and Biotechnology, University of Pisa, Pisa, Italy; 3 Centro Neurolesi “Bonino-Pulejo” IRCCS, Messina, Italy; 4 CNR Institute of Neuroscience, University of Milan, Milan, Italy; 5 Monzino Cardiologic Center IRCCS, Milan, Italy; 6 Department of Neuroscience, University of Turin, Turin, Italy; 7 Institute of Pharmacology and Pharmacognosy, University of Urbino, Urbino, Italy; Chiba University Center for Forensic Mental Health, Japan

## Abstract

Deciphering the mechanisms regulating the generation of new neurons and new oligodendrocytes, the myelinating cells of the central nervous system, is of paramount importance to address new strategies to replace endogenous damaged cells in the adult brain and foster repair in neurodegenerative diseases. Upon brain injury, the extracellular concentrations of nucleotides and cysteinyl-leukotrienes (cysLTs), two families of endogenous signaling molecules, are markedly increased at the site of damage, suggesting that they may act as “danger signals” to alert responses to tissue damage and start repair. Here we show that, in brain telencephalon, GPR17, a recently deorphanized receptor for both uracil nucleotides and cysLTs (e.g., UDP-glucose and LTD_4_), is normally present on neurons and on a subset of parenchymal quiescent oligodendrocyte precursor cells. We also show that induction of brain injury using an established focal ischemia model in the rodent induces profound spatiotemporal-dependent changes of GPR17. In the lesioned area, we observed an early and transient up-regulation of GPR17 in neurons expressing the cellular stress marker heat shock protein 70. Magnetic Resonance Imaging in living mice showed that the *in vivo* pharmacological or biotechnological knock down of GPR17 markedly prevents brain infarct evolution, suggesting GPR17 as a mediator of neuronal death at this early ischemic stage. At later times after ischemia, GPR17 immuno-labeling appeared on microglia/macrophages infiltrating the lesioned area to indicate that GPR17 may also acts as a player in the remodeling of brain circuitries by microglia. At this later stage, parenchymal GPR17^+^ oligodendrocyte progenitors started proliferating in the peri-injured area, suggesting initiation of remyelination. To confirm a specific role for GPR17 in oligodendrocyte differentiation, the *in vitro* exposure of cortical pre-oligodendrocytes to the GPR17 endogenous ligands UDP-glucose and LTD_4_ promoted the expression of myelin basic protein, confirming progression toward mature oligodendrocytes. Thus, GPR17 may act as a “sensor” that is activated upon brain injury on several embryonically distinct cell types, and may play a key role in both inducing neuronal death inside the ischemic core and in orchestrating the local remodeling/repair response. Specifically, we suggest GPR17 as a novel target for therapeutic manipulation to foster repair of demyelinating wounds, the types of lesions that also occur in patients with multiple sclerosis.

## Introduction

Extracellular adenine (ATP, ADP), uracil (UTP, UDP) and sugar nucleotides (e.g., UDP-glucose and UDP-galactose) are universal and phylogenetically ancient signaling molecules involved in many biological processes, from embryogenesis to adult homeostasis [1]. Their actions on target cells are mediated by specific membrane receptors: the seven ligand-gated purinergic P2X channels and the eight G protein-coupled P2Y receptors (the P2Y_1,2,4,6,11,12,13,14_ receptors) [1], [2]. Owing to their involvement in the regulation of many physiological phenomena, dysfunctions of nucleotides and their receptors have been associated to various human diseases, including ischemic/inflammatory conditions [1], [2]. Instead, cysteinyl-leukotrienes (cysLTs, such as LTC_4_, LTD_4_ and LTE_4_) are inflammatory 5-lipoxygenase arachidonic acid mediators [3] with established roles in bronchial asthma [4] acting through the G protein-coupled CysLT_1_ and CysLT_2_ receptors [5].

Recent data highlight the existence of a functional crosstalk between the nucleotide and the cysLT systems in orchestrating inflammatory responses in various disease conditions, including ischemic stroke. Both types of mediators accumulate at the sites of inflammation, and inflammatory cells often co-express both P2Y and CysLT receptors [6]. In rat microglia, the brain immune cells involved in the response to cerebral hypoxia and trauma, the activation of P2Y_1_ and CysLT receptors mediate co-release of nucleotides and cysLTs [7], which might, in turn, contribute to neuroinflammation and neurodegeneration. In the middle cerebral artery occlusion (MCAo) model in the rat, levels of cysLTs in the lesioned cortex were sharply increased 4 h after MCAo and rapidly declined afterwards [8]. In the same experimental model, extracellular concentrations of ATP were instead constantly elevated starting from 20 min after MCAo throughout a 220 min of microdialysis sampling, with a time course similar to that of excitatory amino acids [9]. Thus, at the site of brain injury, neurons and glia are exposed to high concentrations of both extracellular nucleotides and cysLTs, suggesting that these signaling molecules may act as “danger signals” to alert responses to tissue damage and start repair.

We have recently reported that GPR17, a previously orphan G protein-coupled receptor [10] (GPCR) at an intermediate phylogenetic position between P2Y and CysLT receptor families, is a new P2Y receptor specifically activated by both uracil nucleotides (UDP, UDP-glucose and UDP-galactose) and cysLTs (LTD_4_ and LTC_4_) [11]. Consistent with this hybrid pharmacology, activation of GPR17 by uracil nucleotides was counteracted by the P2Y receptor antagonists cangrelor and MRS2179, whereas its activation by cysLTs was antagonized by montelukast and pranlukast, two already marketed CysLT receptor blockers (ibidem). Both the human and the previously unknown rat receptor were found to be highly expressed in organs typically undergoing ischemic damage, i.e., brain, heart and kidney. Moreover, in the MCAo rat model, 48 hours after ischemia induction, GPR17-immunoreactivity was markedly increased in the ipsilateral cortex both within and at the borders of the ischemic infarct, suggesting a potential role in the evolution of ischemic damage. On the other hand, in a recent transcriptome study comparing the gene expression pattern of human *fetal* and *adult* neuroprogenitor cells (NPCs), GPR17 was identified as one of the three genes that were exclusively expressed in adult hippocampal NPCs [12], thus highlighting a potential role in brain repair. Thus, GPR17 may have a differential role in brain response to injury, depending upon whether the receptor is already constitutively expressed by the cell or is induced as a result of brain insult. Moreover, a differential role of GPR17 may also depend on specific phases of brain response after the insult (sequentially, death of irreversibly damaged cells, clearance of dead cells, remodeling and repair).

On this basis, the present study has been specifically aimed at assessing the localization of GPR17 in cells of different lineages (neurons, glia and neural progenitor cells) of the intact mouse brain and its changes at different times after induction of focal cerebral ischemia in the MCAo model.

We show that in the intact brain GPR17 is expressed by neurons and by a subset of adult quiescent parenchymal oligodendrocyte progenitors. Following ischemia, GPR17 is sequentially induced in neurons, microglia/macrophages and adult oligodendrocyte precursor cells, suggesting that it may act as a common regulatory gene mediating response to injury in embryonically-distinct cell types. This indicates GPR17 as a possible target for pharmacological intervention in neurodegeneration processes including cerebral ischemia and demyelinating diseases.

## Results

### Cloning and characterization of mouse GPR17

To study in detail the role of GPR17 in brain injury, the well established model of focal cerebral ischemia in the mouse (MCAo) was chosen. Thus, as a first step, we cloned and characterized the previously uncharacterized murine GPR17 ortholog (GenBank NM_001025381). Mouse GPR17, which is localized on chromosome 18, contains a 1020 base pairs open reading frame (ORF) that encodes for a putative 339 amino acid protein 90 and 98% identical to human and rat GPR17 [11]. Specific external primers were used to amplify cDNA from several mouse tissues. Specific PCR products of the expected mw (1022 bp) were obtained from mouse brain, kidney, heart and skeletal muscle, but not from liver and lung (Figure S1A). The 1022 bp PCR product from mouse brain cDNA was then cloned into a pcDNA3.1/V5-His-TOPO expression vector. The plasmid containing the positive murine GPR17 clone (pcDNA3.1-Gpr17m) was used to transiently transfect 1321N1 human astrocytoma cells, a cell line lacking endogenous P2Y receptors and already utilized for the characterization of human and rat GPR17 [11]. Expression of the transfected receptor was assessed by the appearance of a PCR product of the expected 1022 bp length in cells receiving pcDNA3.1-Gpr17m, whereas no expression of mGPR17 was found in cells transfected with the corresponding empty vector (Figure S1B). To confirm that the receptor transcript is indeed translated into the receptor protein, we utilized a rabbit anti-GPR17 polyclonal antibody that also recognizes human and rat GPR17 [11]. As expected, upon incubation with this antibody, 1321N1 cells transfected with pcDNA3.1-Gpr17m showed a specific immunoreactive signal which, instead, was not detected in cells receiving the corresponding empty vector (Figure S1C). To further confirm the specificity of these results, in the mGPR17-expressing cells, pre-adsorption of the anti-receptor antibody with the neutralizing peptide utilized for immunization completely abolished the GPR17 signal (Figure S1C).

**Figure 1:**
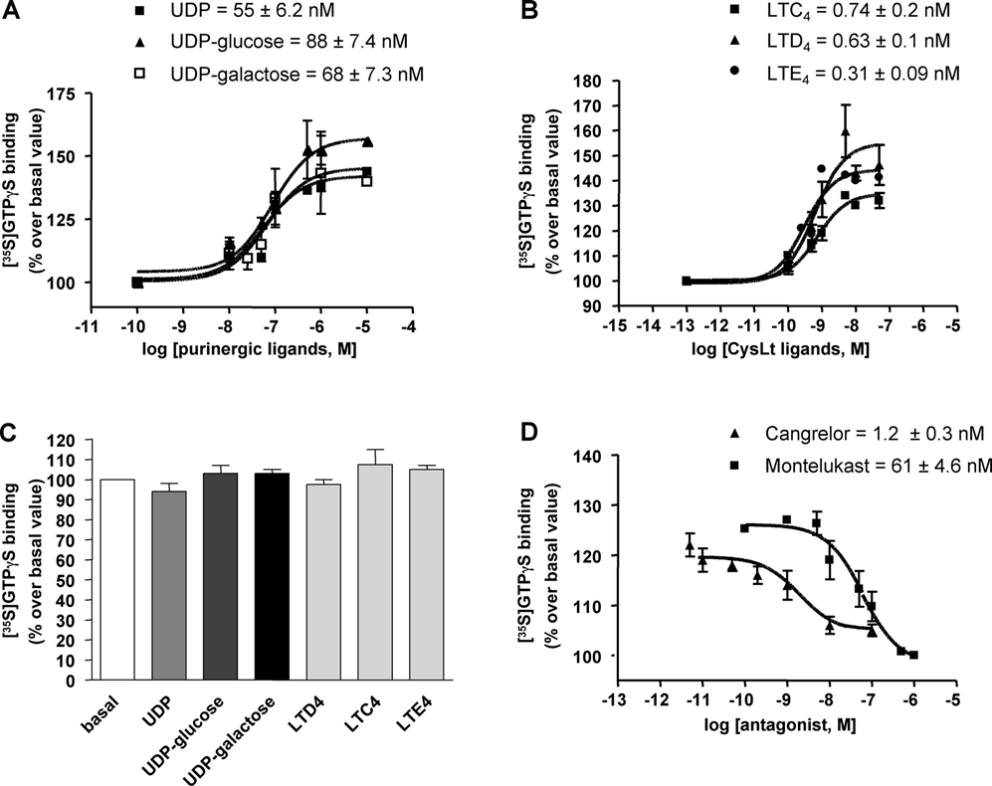
Effect of purinergic and leukotriene ligands on mGPR17 with [^35^S]GTPγS binding. Membrane aliquots (10 µg), obtained from 1321N1 cells transfected with pcDNA3.1-Gpr17m (A,B), were incubated with the indicated concentrations of purinergic (A) or leukotriene (B) agonists. (C) Membranes from cells transfected with the pcDNA3.1 empty vector were incubated with UDP, UDP-glucose or UDP-galactose (all at 10 µM) or with LTD_4_, LTC_4_ and LTE_4_ (all at 50 nM), as indicated. (D) Effect of P2Y or CysLT receptor antagonist on agonist-stimulated [^35^S]GTPγS binding. Membranes from pcDNA3.1-Gpr17m transfected cells were pre-incubated for 10 min with graded montelukast (1 nM-1 µM) or Cangrelor (0.01 nM-100 nM) concentrations and then stimulated with 500 nM UDP or 50 nM LTD_4_, respectively. All data are expressed as percentage of basal [^35^S]GTPγS binding (set to 100%) and represent the mean±SEM of three different experiments each performed in duplicate.

The pharmacological profile of mGPR17 was then assessed. Since stimulation of GPCRs results in increased binding of GTP to G proteins (which can, in turn, be quantified by measuring [^35^S]GTPγS binding to purified membranes; see [13]), GPR17 activation in the heterologous system was determined by testing the increase of [^35^S]GTPγS binding by exogenously-added agonists in membranes from transfected cells [13], [14], [15]. Full concentration-response curves with the uracil nucleotide UDP, the sugar nucleotides UDP-glucose and UDP-galactose and the cysLTs LTC_4_, LTD_4_ and LTE_4_ were performed. In GPR17-expressing cells, UDP, UDP-glucose and UDP-galactose concentration-dependently stimulated GTPγS binding with EC_50_ values of 55±6.2, 88±7.4 and 68±7.3 nM, respectively (Figure 1A). All tested leukotriene derivatives (i.e., LTC_4_, LTD_4_, and LTE_4_) were able to stimulate receptor-G protein coupling with a similar potency, with estimated EC_50_ values of 0.74±0.2 nM for LTC_4_, 0.63±0.1 nM for LTD_4_ and 0.31±0.09 nM for LTE_4_ (Figure 1B). None of these agonists, tested at the highest concentration utilized in Figure 1A and 1B, had any effect in cells transfected with the pcDNA3.1 empty vector (Figure 1C). The ability of some known purinergic and cysLT antagonists to counteract agonist-induced GTPγS binding stimulation in cells expressing mGPR17 was also assessed. Cangrelor, a P2Y_12/13_ antagonist, which displayed a high affinity towards human and rat GPR17 [11] concentration-dependently inhibited GTPγS binding stimulated by UDP with an IC_50_ value of 1.2±0.3 nM. In a similar way, the CysLT1 antagonist montelukast concentration-dependently inhibited GTPγS binding stimulated by LTD_4_, with an IC_50_ value of 61±4.6 nM (Figure 1D).

Globally, these data characterize the previously unknown mouse GPR17 receptor, demonstrate that this receptor is indeed expressed at high levels in various organs (including brain) and that, in a similar way to the already characterized human and rat receptors, this receptor can be dually activated by two distinct classes of endogenous ligands, uracil nucleotides and cysLTs, which are both released in great amounts in the injured brain. Moreover, they also show that the mouse receptor more closely resembles the response profile of the human receptor [11].

### In brain, GPR17 is normally expressed in neurons and in a subset of adult neural precursor cells

**Figure 2:**
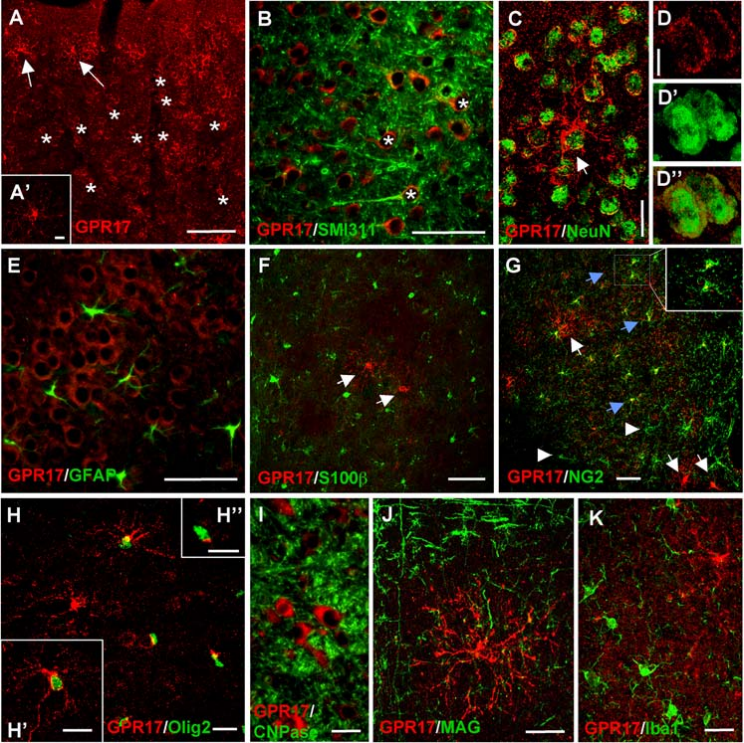
Cellular localization of GPR17 in the intact mouse brain. Double fluorescence labeling of mouse brain coronal sections with anti-GPR17 antibody (red fluorescence) and markers for different cell lineages (green fluorescence). (A) In the intact cortex, GPR17 expression is detected in large round cells present at high density (examples are illustrated by asterisks) and in sparse ramified cells (arrows and A′ inset). Virtually all GPR17^+^ large cell bodies display co-expression of the neuronal proteins SMI311 (B) or NeuN (C), demonstrating their neuronal identity. D panels show higher magnification images of two neurons showing immunopositivity for both GPR17 (red channel, D), and NeuN (green channel, D′); (D″) merge of the two fluorescence channels. In cortex, we found no co-localization of GPR17 with the astroglial markers GFAP (E) and S100β (F, arrows indicate GPR17^+^ cells); several ramified GPR17-labeled cells instead co-stained for either NG2 (blue arrows in G) or Olig2 (H, H′), which are both pre-oligodendrocyte markers. However, in the case of NG2, only a partial co-localization was found, since some cells only stained for NG2 (arrowheads in G) and some others only stained for GPR17 (white arrows in G, H). Co-localization was much higher in the case of GPR17 and Olig2 (see text for quantification). In some cases, NG2^+^ or Olig2^+^ cells show immunoreactivity for GPR17 in discrete cell body compartments (inset in G and H″, respectively; for details, see text). I and J depict no co-localization of GPR17 with myelin-related proteins (CNPase in I and MAG in J). Some “resting” microglial cells were also found in cortex, as suggested by staining with the specific marker Iba1 (K): in the intact brain, none of these cells expressed GPR17. Scale bars: A: 25 µm; A′, D, D′, D″, H′, H″: 10 µm; B, E, F, G: 50 µm; H, I, J, K: 20 µm.

We then utilized the specific anti-GPR17 antibody to determine the cellular localization of GPR17 in control mouse brain. Coronal sections taken at different brain levels were immuno-labeled with the anti-receptor antibody in parallel with markers of neuronal or glial cells. In the intact cerebral cortex, immunohistochemical studies with a high sensitivity amplification kit revealed GPR17 expression in two distinct types of cells. One cell type had neuronal morphology (see asterisks in Figure 2A): the neuronal nature of these GPR17-positive (GPR17^+^) cells was confirmed by co-staining with the neuronal specific markers SMI-311 (Figure 2B) and NeuN (Figure 2C). Higher magnification analysis of these cells suggested that, as expected for a GPCR, GPR17 was selectively expressed in the plasma-membrane of NeuN^+^ neurons (Figure 2D, 2D′, 2D″). Conversely, no co-localization was found with cortical astrocytes, as shown by labeling with the specific markers GFAP (Figure 2E) and S100β (Figure 2F). The other cell type expressing GPR17 displayed small cell bodies with fine radiating processes (see arrows in Figure 2A, 2A′ inset, and arrows in Figure 2F). These cells were dispersed in both gray (Figure 2A) and white matter (not shown) and were reminiscent of precursor cells [16]. To investigate the nature of the highly branched GPR17^+^ cells, we performed staining for the pre-oligodendroglial specific proteoglycan NG2 [17]. Double-staining experiments revealed a heterogeneous population of ramified cells. In particular, 3 distinct phenotypes of GPR17^+^ ramified cells were found: a first cell type was characterized by exclusive expression of NG2 (arrowheads in Figure 2G), a second by exclusive expression of GPR17 (white arrows in Figure 2G) while a third cell type co-expressed both markers (blue arrows in Figure 2G and inset). Quantitative analysis of these cells showed that about 1/4 of the ramified GPR17^+^ cells also displayed NG2 staining. Co-expression with markers of pre-oligodendrocytes was further confirmed using an antibody against the transcription factor Olig2, a key regulator of oligodendrocyte development [17]. In this case, a much higher number of GPR17^+^ cells were also positive for Olig2. Quantitative fluorescence microscopy analysis of full hemisphere sections in 3 animals showed that 59.18±5.46% of the ramified GPR17^+^ cells indeed also co-expressed Olig2 in their nuclei (Figure 2H and 2H′; a mean of 680±52.2 GPR17^+^ cells were counted/animal; of these, 403±49.7 also positively stained for Olig2).

Interestingly, in some Olig2 or NG2-labeled cells, GPR17 immunoreactivity displayed an asymmetrical subcellular localization, suggesting GPR17 accumulation in the Golgi apparatus (Figure 2H″ and inset in Figure 2G). This specific staining pattern may represent a well defined GPR17 maturation stage prior to distribution of the receptor to the plasma-membrane. The branched GPR17^+^ cells did not incorporate BrdU in their nuclei, at least under the conditions of BrdU treatment utilized here (data not shown), suggesting that, in the intact brain, GPR17 decorates a subset of quiescent non-proliferating parenchymal oligodendrocyte precursors. Furthermore, no colocalization of GPR17 was found with more mature myelinating oligodendroglial markers, as shown by lack of double-staining with either CNPase (Figure 2I), myelin-associated glycoprotein MAG (Figure 2J) and myelin basic protein MBP or APC (data not shown), suggesting that GPR17 may be expressed at a specific stage of oligodendroglial differentiation. Finally, no GPR17 co-expression was found in resting microglia detected by anti-Iba1 staining in the intact brain (Figure 2K) (however, see: “Expression pattern of GPR17 during ischemia evolution”).

GPR17^+^ ramified cells were also found in corpus callosum: most of these cells co-stained for Olig2 but not with NG2 and did not seem to proliferate under resting conditions (not shown). Interestingly, in c. striatum, GPR17^+^ precursor cells were not positive for MBP, but were often found physically associated to or inside myelinating fibers (Figure S2).

**Figure 3:**
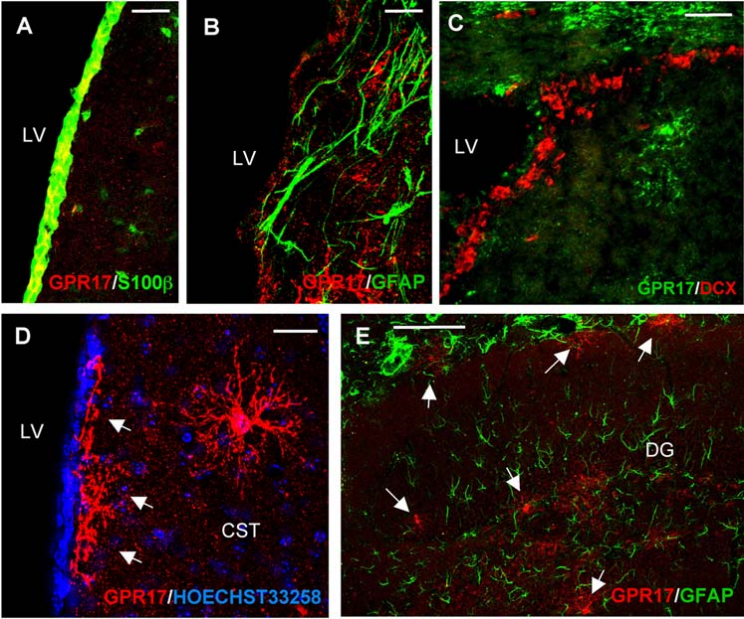
GPR17 expression in the mouse germinative zones. Micrographs depicting GPR17 staining in coronal sections of the intact anterior forebrain. (A) In the walls of the lateral ventricles (LV), GPR17 (red fluorescence) is highly expressed in ependymal cells, which are in direct contact with ventricle cavities and also express the S100β protein (green fluorescence). Cells co-expressing both GPR17 and S100β in this cell layer are indicated in yellow. (B, C) Specific GPR17 staining (red fluorescence) is also found in the subependymal layer of LV. (B): several of these cells show a morphology that is reminiscent of the GPR17^+^ oligodendrocyte precursors found in brain parenchyma (see also Figure 2 and panel D). These GPR17^+^ cells do not co-stain for the astrocytic marker GFAP (green fluorescence in B), that is also specifically expressed by multipotent adult stem cells. (C) As expected, in the subependymal layer of lateral ventricles, several double-cortin (DCX)^+^ neuronal precursors are evident (red fluorescence); however, these cells do not co-express GPR17 (here shown in green fluorescence). (D) An additional image of GPR17^+^ ramified precursors (red fluorescence) in LV subependymal layer (arrows) and in c. striatum (CST) of brain parenchyma (Hoechst 33258 staining of cell nuclei is also shown in blue). GPR17^+^ cells in the subependymal layer were often found to protrude their processes in the ventricular cavity. (E) Double fluorescence image showing expression of GPR17 (red fluorescence) in ramified cells in the hippocampus (DG: dentate gyrus). Also in this brain area, GPR17 immunoreactivity does not co-localize with GFAP (green fluorescence). Scale bars: A–C 50 µm; D 10 µm; E 100 µm.

Based on previous data demonstrating that GPR17 is one of the 3 key genes expressed in human *adult* NPCs [12], we also looked at the presence of GPR17^+^ cells in classical mouse brain neurogenic areas, i.e., the subventricular zone (SVZ) of lateral ventricles (LV) and the dentate gyrus (DG) of hippocampus. The first layer of brain ventricles wall consists of ependymal cells, i.e., radial glia-derived cells that intensively stain for the astrocyte marker S100β [18] (green fluorescence in Figure 3A). Several of these cells were also immunoreactive for GPR17 (red fluorescence in Figure 3A; cells expressing both S100β and GPR17 are in yellow). GPR17^+^ ramified cells with a morphology very similar to that of the parenchymal precursors described above were also detected immediately below the ependyma as well as in more profound layers of the subependymal zone (Figure 3B–D). These cells never co-stained with either the stem cell marker nestin (not shown), GFAP (Figure 3B) or the neuronal precursor marker double-cortin (DCX) (Figure 3D), suggesting that GPR17 is not expressed by GFAP^+^ multipotent stem cells or by DCX^+^ neuronal precursors [19], [20], [21]. Highly branched GPR17^+^ cells were also found in the subgranular layer of the hippocampus (Figure 3E); also here, no colocalization of GPR17 with the adult neural stem cell marker GFAP was found. None of these GPR17^+^ precursors proliferated under normal conditions.

Globally, the data described above show that, in the intact mouse brain, GPR17 is expressed by neurons and by a subset of precursor cells in both parenchyma and neurogenic areas. In the parenchyma, GPR17 segregates to a subset of NG2^+^/Olig2^+^ precursor cells that never proliferated under control conditions, suggesting that there are quiescent pre-oligodendrocytes. In the neurogenic areas, GPR17 decorates a subset of non-proliferating, nestin^−^, GFAP^−^ and DCX^−^ cells of yet undefined nature.

### Activation of GPR17 *in vitro* by the endogenous agonists UDP-glucose and LTD_4_ promotes pre-oligodendrocyte differentiation to mature myelinating cells

**Figure 4:**
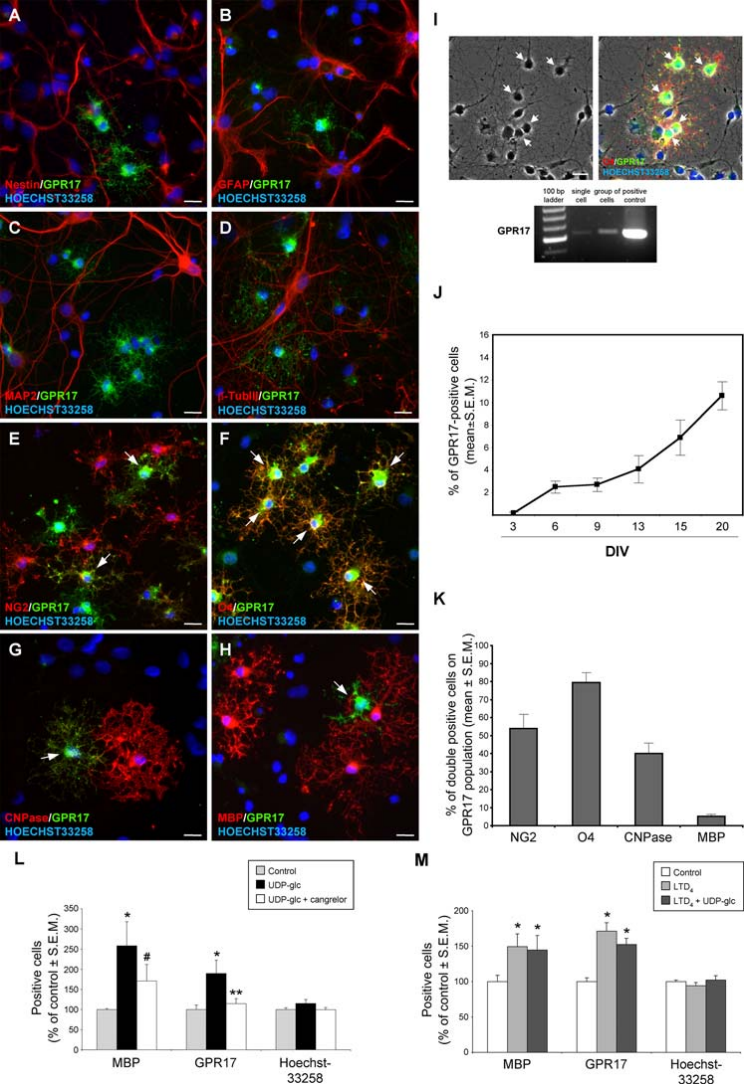
Presence and activation of GPR17 in oligodendrocyte precursor cells of primary cortical cultures maintained *in vitro*. Several GPR17^+^ ramified cells (green fluorescence) reminiscent of the precursors found *in vivo* were consistently found in primary neuron/glia cortical cultures. Micrographs (A–H) show their phenotype characterization. These cells (green fluorescence) did not co-express the stem cell marker nestin (A), or the astrocytic marker GFAP (B), or the neuronal markers MAP2 (C) and β-TubIII (D), but were instead positive for several pre-oligodendrocyte markers, such as NG2 (arrows in E) and O4 (arrows in F), which appear quite early during oligodendrocyte differentiation. Colocalization of GPR17 with these markers is shown in yellow-orange cells. A lower number of GPR17^+^ cells also expressed CNPase (arrow in G) and very few costained for MBP (arrow in H), which are markers of more mature oligodendroglia (for more details, see text). In blue, Hoechst 33258 stained nuclei. Single cell RT-PCR was used to confirm expression of GPR17 in oligodendroglial-like cells (I). For this analysis, cells were picked up from living cultures using cell morphology as a guide at the light microscope. Left micrograph: an example of a brightfield showing cells (arrows) with oligodendrocyte morphology. These cells can be distinguished from neurons based on their size (approximately 1/3 the size of neurons), phase-dark cell bodies and presence of fine ramifications. The oligodendrocyte nature of cells with this morphology was confirmed by co-immunostaining for O4 and GPR17 at a subsequent time after culture fixing (arrows in right micrograph). By following this method, one living cell or a small number of cells (up to 10) were sucked from living cultures (see MATERIALS AND METHODS) and processed for RT-PCR analysis. Significant GPR17 expression was detected in both the single-cell and the 10-cell samples, as indicated in the PCR panel. Rat cortex was utilized as a positive control (see last lane). Scale bars: 15 µm. In these primary cultures, the number of GPR17^+^ cells spontaneously increased as a function of time (J); DIV: days *in vitro*. (K) Quantification of the percentage of GPR17^+^ cells that also co-express the indicated oligodendrocyte markers. Total number (n) of GPR17^+^ cells counted from two different coverslips for each preparation: for NG2, n = 3502; for O4, n = 2685; for CNPase, n = 1738; for MBP, n = 3615. Data were obtained from five different primary culture preparations. (L) Exposure of cultures to maximal (100 µM) UDP-glucose (UDP-glc) concentrations for 72 h induced a highly significant increase in the number of mature MBP^+^ oligodendroglial cells. The number of GPR17^+^ cells was also increased. Both effects were partially counteracted in the presence of the P2Y receptor antagonist cangrelor. (M) Exposure of cultures to maximal (100 nM) LTD_4_ concentrations for 72 h also induced a significant increase in the number of mature MBP^+^ oligodendroglial cells and of GPR17^+^ cells. No synergistic nor additive effects were found when both agonists were combined together. There were no significant changes in the total number of cells in culture during the 72 h treatment, as shown by labeling of cells nuclei with Hoechst 33258. *p<0.05 compared to control; ** p<0.05 with respect to UDP-glc and not different from control; # p = 0.15 with respect to control and P = 0.245 with respect to UDP-glc, one-way analysis of variance (ANOVA).

To gain more information on the role of GPR17 in pre-oligodendrocyte precursors, we characterized the behaviour of these cells in mixed neuron/glia cortical cultures, where many GPR17^+^ cells were found (Figure 4A–H). These cells displayed a very typical highly ramified morphology resembling that of the precursor cells found *in vivo*. As also observed in the intact brain for the parenchymal precursors, these GPR17^+^ cells never expressed nestin (Figure 4A), GFAP (Figure 4B), MAP2 (Figure 4C) or β–TubIII (Figure 4D), suggesting that they are not stem cells, nor astrocytes or neurons. To investigate in detail whether GPR17 is specifically expressed by pre-oligodendrocytes, we performed double staining experiments with a set of markers identifying different stages of differentiation, i.e., NG2 (oligodendrocyte precursor), O4 and CNPase (pre-oligodendrocytes, immature/mature cells), and MBP (mature oligodendrocytes) [22], [23]. GPR17^+^ cells were found to co-express either NG2 (arrows in Figure 4E), Olig2 (not shown), O4 (arrows in Figure 4F), or, to a lesser extent, CNPase (Figure 4G), confirming that they are indeed oligodendrocyte precursors. Conversely, very few cells co-expressed the mature oligodendrocyte marker MBP (Figure 4H; see also below). Expression of GPR17 in oligodendrocyte precursors was also confirmed by single-cell PCR. For this analysis, one cell or a small group of cells (up to 10) were picked up from living cultures using cell morphology as a guide at the light microscope (see arrows in Figure 4I). Receptor expression was detected in both the single-cell and the 10-cell samples, as indicated in the PCR panel of Figure 4I, where rat cortex was utilized as a positive control.

The number of the GPR17^+^ precursors constantly increased with time in culture (Figure 4J). A quantification of the percentage of the GPR17^+^ cells at various oligodendrocyte differentiation stages in 10-day old cultures is shown in Figure 4K. A prevalence of GPR17^+^ cells expressing early oligodendroglial markers such as NG2 or O4, was constantly found. No significant relative changes of this distribution was observed with time in culture, at least up to day 20 (data not shown).

In order to verify whether GPR17 in cultured precursors is functional and whether its activation can promote cell progression along their differentiation pathway, cultures were treated with UDP-glucose, which selectively binds to this receptor and activates it [11]. Exposure of cultures to a maximal concentration (100 µM) of UDP-glucose for 72 h did not have any effect on cell viability, as demonstrated with propidium iodide (PI) that does not enter viable intact cells and can only label the nuclei of cells with damaged permeabilized membrane. In control cultures, PI labeled a small percentage of cells (16.3±3.5%, n = 4). This low percentage of cell death, which is reasonably expected for a primary *in vitro* culture, was not different in UDP-glucose treated cells (13.7±1.9%, n = 4). UDP-glucose treatment instead resulted in a significant increase of the number of GPR17^+^ precursor cells, suggesting that activation of the receptor may itself promote receptor expression, and, more important, it markedly increased the number of mature MBP^+^ oligodendroglial cells (Figure 4L). Both effects were partially counteracted by the P2Y receptor antagonist cangrelor (ibidem). Exposure to a maximal concentration (100 nM) of LTD_4_ also significantly increased both the number of GPR17^+^ and mature MBP^+^ oligodendroglial cells (Figure 4M). No synergistic effects were observed when cells were treated with a combined protocol (Figure 4M). The total number of cells in culture did not significantly increase during the 72 h treatment period with pharmacological agents, as suggested by labeling of cell nuclei with Hoechst 33258 (Figure 4L and 4M), to support the conclusion that, in these cultures, activation of GPR17 by its endogenous agonists is actually inducing the *in vitro* maturation of already existing oligodendrocyte precursors.

Thus, GPR17 is already functional in pre-oligodendrocytes and its activation promotes the progression of these cells along their differentiation pathway.

### Expression pattern of GPR17 during ischemia evolution

**Figure 5:**
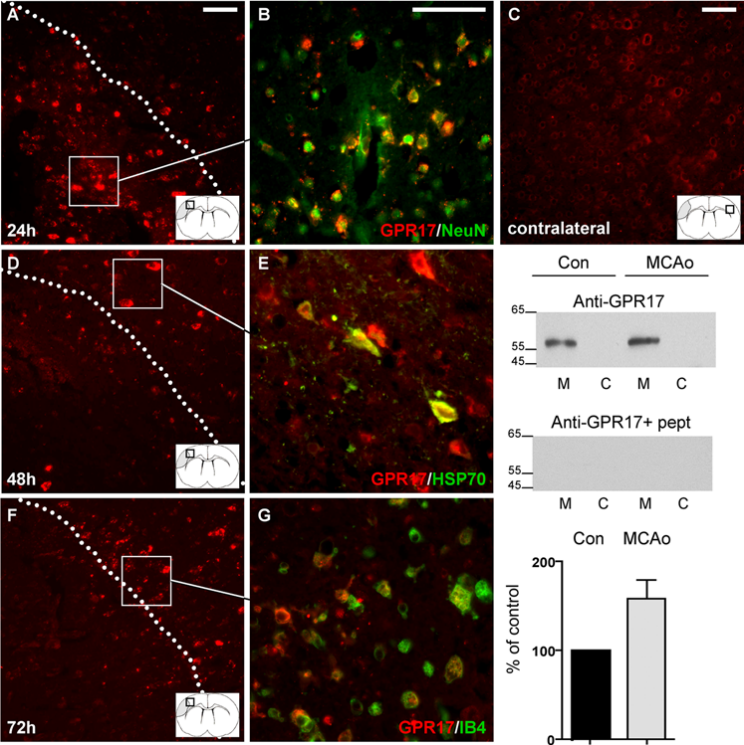
Time course of GPR17 expression after MCAo in mice. Micrographs show coronal brain slices obtained from mice 24, 48, 72 hours after MCAo. Brain drawings in insets show the areas (squares) where micrographs were taken. (A) Twenty-four h after MCAo, immunohistochemistry with an anti-GPR17 antibody (red fluorescence) show a marked up-regulation of the receptor within the ischemic lesioned area (the region delimited by the dotted line). Basal expression of GPR17 in the corresponding contralateral area is shown in C for comparison. (B) Cells showing marked GPR17 expression inside the lesioned area also express the specific neuronal marker NeuN, confirming that these cells are indeed neurons. (D) Forty-eight h after MCAo, GPR17^+^ neurons inside the lesioned area are markedly reduced, suggesting induction of cell death. At this time point, a marked up-regulation of GPR17 appears in neurons at the border of the lesion. (E) These cells co-localize with the neuronal damage marker HSP70 (see text for more details). Up-regulation of GPR17 in the ischemic area is also confirmed by western blot analysis in membrane (M) and cytosolic (C) fractions from intact contralateral (Con) and ipsilateral ischemic (MCAo) cortex of mice (right panel). GPR17 is identified with an anti-GPR17 antibody as an immunoreactive protein band with an approximate 50 kDa molecular weight; as expected for a membrane receptor, this band is present in membranes but not in cytosolic fractions. To confirm blot specificity, the 50 kDa protein band is abolished in the presence of the neutralizing peptide (indicated as+pept). A marked increase of the intensity of this band is found after MCAo (the quantification of this increase is shown in histograms as % of control set to 100%; results from 5 experiments). Forty-eight h (not shown) and seventy-two h after MCAo (F), there is a marked increase of GPR17^+^ cells at the border of the lesioned area. (G) These cells are not neurons but they are microglia/macrophages infiltrating the ischemic area, since most of them also positively stain for IB4, a microglia/macrophage marker (see also text). These GPR17^+^/IB4^+^ cells are indeed found inside the lesioned area one week after MCAo (data not shown). Scale bars: 50 µm.

To characterize the changes of GPR17 occurring during ischemic damage, mouse brains were processed for immunohistochemistry at 24, 48, 72 hours and 1 week after MCAo. Within the lesioned area, a strong up-regulation of GPR17 was observed 24 h after MCAo (Figure 5A) with respect to the corresponding unlesioned area of the contralateral cortex (Figure 5C). Inside the injured area, most of the GPR17^+^ cells co-localized with the neuronal marker NeuN (Figure 5B), suggesting that receptor up-regulation indeed occurred in neurons. Forty-eight hours after MCAo, a dramatic decrease of both the number of neuronal cells and of GPR17 immunoreactivity was found inside the lesion, suggesting that neurons over-expressing GPR17 at 24 h had undergone cell death (Figure 5D). Several shrunk cells overexpressing GPR17 and resembling damaged neurons were also found at the borders of the ischemic infarct (Figure 5D). Some of these cells also positively stained for NeuN (data not shown) and for the chaperon protein HSP70 (Figure 5E), which is known to be up-regulated in neurons upon damage [24]. Western blot analysis of GPR17 performed in membrane (M) and cytosolic (C) fractions confirmed increased expression of a membrane protein band of approximately 50 kDa (likely corresponding to a glycosylated form of the receptor) in the lesioned hemisphere (MCAo) compared to the intact contralateral (Con) side (Figure 5, right panel). As expected for a membrane receptor, no specific signal for the anti-GPR17 antibody was detected in cytosolic fractions (ibidem). To confirm that this band is indeed GPR17, immunolabeling was abolished in the presence of the neutralizing peptide. A quantification of the MCAo-induced up-regulation of GPR17 is shown in histograms. Globally, these findings suggest that, upon damage, GPR17 is up-regulated in injured neurons and that this up-regulation may be causally related to cell death. At this stage of damage development, GFAP^+^ activated astrocytes indicating astrogliosis were also visible at the borders of the injured area, but none of these cells expressed GPR17 (data not shown).

Starting from 48 h and highly evident seventy-two h after MCAo, a marked expression of GPR17 was found at the boundaries of the lesioned area (Figure 5F), but this time immunoreactivity was associated to cells also expressing IB4, a marker of activated microglia/macrophages (Figure 5G). Under the staining conditions utilized here, no specific IB4 labeling was detected in the intact contralateral side (data not shown). One week after MCAo, GPR17 immunopositivity was again increased within the damaged area: also these cells were all IB4^+^, suggesting that they are indeed the macrophages/microglia found at the lesion borders at day 3, that were now infiltrating the lesioned area (data not shown). These data show that after up-regulation in neurons, there is a second wave of GPR17 induction in microglia/macrophages as soon as these cells are activated and recruited toward the lesioned area as a consequence of damage.

**Figure 6:**
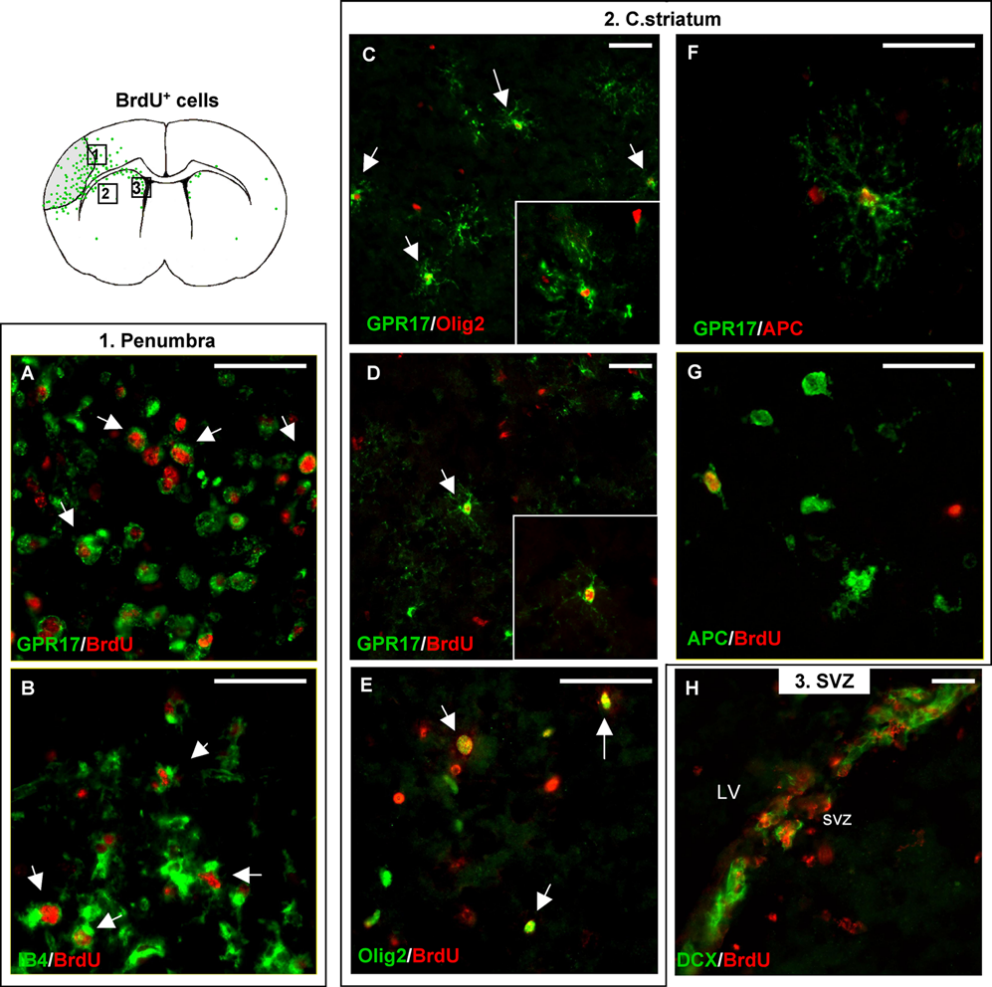
Characterization of proliferating cells in adult mouse brain after induction of MCAo. To evaluate injury-induced cell proliferation, BrdU (50 mg/Kg) was administered twice a day for 3 days to ischemic mice starting from the afternoon of MCAo. Animals were sacrified 4 h after the last BrdU injection and coronal brain slices prepared for immunohistochemical analysis (for further details, see Materials and Methods). Seventy-two h after MCAo, a marked increase of BrdU^+^ cells is found in the ipsilateral lesioned hemisphere with respect to the contralateral unlesioned side (compare green dots in brain's drawing). Many of these cells are also GPR17^+^. Two main types of BrdU^+^/GPR17^+^ cells are found. (A, B) A first, small globular cell type is mainly found in the peri-lesioned area (indicated as 1 in brain's drawing); these cells also markedly stain for IB4 and are indeed the microglia/macrophages about to infiltrate the ischemic lesion already described in Figure 5. (C–E) A second, ramified BrdU^+^/GPR17^+^ cell type (D) reminiscent of the parenchymal Olig2^+^ pre-oligodendrocytes (C, E; see also text) already described in the intact brain (Figure 2) is consistently found in the c. striatum (indicated as 2 in brain drawing) as well as in regions closer to the lesioned area (not shown). The number of these cells was significantly higher in the ipsilateral with respect to the unlesioned contralateral side (see text for more details). (F, G) Some of these proliferating precursor cells also positively stained for APC, a marker of mature oligodendroglia, suggesting initiation of re-myelination. (H) As expected, in the ispilateral SVZ (indicated as 3 in brain drawing), a marked increase of double-positive BrdU^+^/DCX^+^ cells is found with respect to the unlesioned hemisphere. However, none of these cells expressed GPR17 (not shown). Scale bars: 50 µm.

Based on the demonstration of GPR17 on NPCs [12] (Figure 3) and on the finding that ischemic damage induces proliferation of adult NPCs in brain's neurogenic areas and parenchyma [25], we investigated whether cells expressing GPR17 also underwent proliferation after MCAo, as assessed *in vivo* by BrdU administration. As shown in Figure 6, 72 h after MCAo, a marked increase in the number of proliferating cells was found in the lesioned ipsilateral hemisphere with respect to the corresponding unlesioned side (compare green dots in brain's schematic drawing). Two main types of BrdU^+^/GPR17^+^ cells were found in the ischemic side. The first cells were mainly found at the borders of the injured area (indicated as 1 in brain's drawing) and appeared as small globular cells (Figure 6A) which markedly stained for IB4 (Figure 6B). So, these cells are the microglia/macrophages about to infiltrate the lesioned tissue, already shown in Figure 5. The morphology of the second proliferating cell type very closely resembled the parenchymal highly branched pre-oligodendrocytes already described in Figure 2 (see also Figure 6C) and positively stained for both GPR17 (Figure 5D) and Olig2 (Figure 5E). These parenchymal branched GPR17^+^ precursors were consistently found in the c. striatum (indicated as 2 in brain drawing) as well as in regions closer to the lesion borders. Quantification of the GPR17^+^ precursor cells in 3 animals revealed a 24.07±5.23% increase in number with respect to the unlesioned contralateral side (total number of cells counted: 2542 and 2039 in the ischemic versus the contralateral side). At variance from the intact brain, in the injured brain, some of these precursor cells also positively stained for APC, a marker of mature oligodendroglia (Figure 6F and 6G). As expected, brain injury also induced the activation of precursor cells in the SVZ (indicated as 3 in brain drawing), where many BrdU^+^/DCX^+^ cells started proliferating (Figure 6H). However, no changes in GPR17^+^ cells were found in this neurogenic area (not shown) at least at this time frame after MCAo.

Globally, these data show that induction of brain damage is associated to activation of GPR17. Very early (24 h) after the insult, GPR17 is transiently upregulated in neurons (which already express this receptor under physiological conditions) in parallel with the appearance of the cellular stress marker HSP70. GPR17 labeling in the lesioned area decreases as cell death progresses. Starting from 48–72 h after ischemia, GPR17 immuno-labeling appears on microglia/macrophages infiltrating the injured area. At this stage, a subset of GPR17^+^ oligodendrocyte progenitors in the peri-lesion area start to proliferate and to express mature oligodendroglia markers, suggesting differentiation to mature cells.

### The *in vivo* knock down of GPR17 markedly attenuates brain ischemic damage in mice

**Figure 7:**
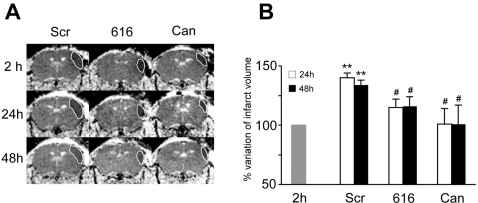
Effect of the *in vivo* knock down of GPR17, by either a biotechnological or a pharmacological strategy, on brain infarct size evolution, as determined by MRI. (A) Representative Tr(D) images of coronal brain sections from ischemic mice treated with either an anti-sense oligonucleotide specifically designed to knock down GPR17 (616) or Cangrelor (Can), in comparison with animals treated with a “scrambled” randomly designed oligonucleotide (Scr). Images were taken from the same animals at 2, 24 and 48 h after MCAo. Dotted lines indicate the extension of the ischemic infarct. (B) Quantitative analysis of infarct size volume at 24 and 48 hours after MCAo in mice receiving Scr, 616 or Can (mean of 6 animals/group). Data are expressed as percentage variation of infarct volume at 24 and 48 h after MCAo compared to 2 h considered as 100% (see also MATERIALS AND METHODS). There were no statistically significant differences between groups in the ischemic infarct volumes at 2 h (Src = 23.4±7.47; 616 = 23.9±7.2; Can = 16.4±4.23 mm^3^). **P<0.001 versus 2 h; # P<0.05 versus corresponding Scr animals.

The data shown above suggest that GPR17 may represent a “sensor” of damage activated very early during ischemia induction and contributing to cell death. If this were true, its inhibition during MCAo should contrast cell death inside the ischemic core and attenuate the evolution of ischemic damage. To address this issue, 6 mice received an anti-sense oligonucleotide (616) specifically designed to knock down this receptor *in vivo*. Animals were injected intracerebroventricularly 20 min and 24 h after MCAo. This biotechnological strategy [26] has been proven to very efficiently downregulate other GPCRs in the brain [27], [28] and has been already successfully utilized by us in ischemic rats [11]. Another group of 6 animals received in parallel a “scrambled” oligonucleotide randomly generated on the basis of the nucleotide sequence of 616. This group of animals was utilized as a control group in this set of experiments. Finally, another group of 6 animals received Cangrelor, that, although not selective for GPR17 (see “Cloning and characterization of mouse GPR17”), has been shown by us to potently inhibit GPR17 in both the rat [11] and in the mouse (the present study). The evolution of ischemic damage was followed longitudinally in living animals by Magnetic Resonance Imaging (MRI) at 2, 24 and 48 h after MCAo. There were no changes between groups in the mean brain ischemic volume at 2 h after MCAo (see Figure 7 legend). In the lesioned hemispheres of animals receiving the scrambled oligonucleotide, as expected, brain infarct volume increased dramatically between 2 and 48 h. Conversely, in animals treated with 616, progression of ischemic damage at 24 and 48 h was markedly reduced, and, in the case of Cangrelor, totally abolished (see Figure 7A for representative images and Figure 7B for quantification and statistical analysis).

Bioinformatic analysis showed that 616, that effectively reduced brain infarct evolution, was not homologous to any other GPCR (see MATERIALS AND METHODS). This strongly indicates that GPR17 is indeed the specific target of 616, thus confirming a specific role for this receptor in the changes associated to ischemic damage development.

## Discussion

### GPR17: a receptor with a dual role in both neuronal death and in brain repair?

We have previously suggested that an up-regulation of the hybrid nucleotide/cysteinyl-leukotriene receptor GPR17 in the ischemic brain may contribute to the evolution of damage [11]. A causal link between up-regulation of GPR17 and extent of brain damage was suggested by data showing that inhibition of GPR17 by either pharmacological agents or by an antisense-oligonucleotide strategy markedly attenuated cell death (ibidem). However, GPR17 has also been reported to be one of the 3 key genes expressed in human adult neuroprogenitor cells [12], which would instead suggest a potential role in brain repair. These apparently conflicting results suggest that GPR17 might play differential roles in different cell types and/or under specific pathophysiological conditions; for example, it may well be that, upon injury, GPR17 is induced in distinct cell types depending upon specific phases of damage development, remodeling and repair.

On this basis, the present study was purposely undertaken to (i) define the cellular localization of GPR17 in the intact mouse brain and shed light on its potential roles under physiological conditions; and (ii), to define possible changes of this receptor in the cell types that participate to brain damage and repair in a well-established focal ischemia model such as the MCAo model in the mouse.

### Mouse GPR17 closely reproduces the functional features of human GPR17

As a first step to these aims, we cloned and characterized the previously unknown mouse ortholog of GPR17 and compared its pharmacology with that of the already known human receptor [11]. Mouse GPR17 is 90% identical to the human receptor and, importantly, its response profile to ligands is closer to that of the human receptor with respect to the rat receptor. Despite some differences in the affinities of UDP and UDP-glucose, which were more potent at the mouse compared to the human receptor, potencies of cysLTs were in the same range. Moreover, while the endogenous ligand UDP-galactose is totally ineffective at rat GPR17, this nucleotide significantly activates both the human [11] and the mouse receptor (the present study). Finally, for antagonists, while there are significant differences between man and rat, the P2Y and CysLT blockers cangrelor and montelukast display almost identical antagonistic activities at both human and mouse GPR17 ([11]; Figure 1). Therefore, globally, the mouse receptor more closely reproduces the features of the human receptor, thus validating the mouse MCAo model utilized here as the most appropriate experimental model to assess the role of GPR17 in brain injury.

### GPR17 as a new player in oligodendrocyte differentiation

Immunohistochemical studies with a specific, in-house made anti-GPR17 antibody showed that, in the intact mouse telencephalon, GPR17 is present on both neurons and in a subpopulation of ramified non proliferating Olig2^+^ or NG2^+^ precursor cells dispersed in both gray and white matter. In particular, the number of ramified precursors expressing both Olig2 and GPR17 is very high, to suggest that Olig2, which is a transcriptional factor, may drive the expression of GPR17 in these cells. By bioinformatic analysis, we indeed found 14 potential Olig2-responsive elements in the upstream region of the GPR17 gene. These responsive elements can bind to either Olig2 itself or to other members of the Olig2 transcription factor family, such as Atoh1, Neurod1, Neurod2, Neurod4, Neurod6, Neurog1, Neurog2, Neurog3, TGF12 and TGF3 (Figure S3). In both the human, rat and mouse GPR17 upstream regions, 6 phylogenetically conserved domains were found: two of them (indicated as E-Box 1 and E-Box 2 in Figure S3) are particularly interesting, since they are identical in the 3 species and contain 2 responsive elements for members of the Olig2 transcription factor family.

In the intact brain parenchyma, none of the GPR17^+^ precursor cells expressed either GFAP, or DCX or mature oligodendrocyte/myelination markers, suggesting that GPR17 specifically decorates mature neurons and quiescent pre-oligodendrocytes. Interestingly, in c. striatum, immature GPR17^+^/MBP-negative precursor cells were often found physically associated to or inside myelin tracts, which have been previously shown to also contain immature Olig2^+^ cells [29]. These GPR17^+^ cells may represent a source of pre-oligodendrocytes able to turn into myelinating cells when necessary; alternatively, these cells could play a role in the local trophic control of myelination. To confirm the link between GPR17 and the oligodendroglial lineage, highly ramified GPR17^+^ precursors specifically expressing markers identifying different stages of oligodendrocyte differentiation are also present in primary cultures obtained from cortical parenchyma. In a similar way to what observed *in vivo*, under control conditions, these cells are not positive for either stem cell, neuronal or glial markers, and very few of them express MBP, a marker of mature oligodendroglia. This suggests that GPR17 expression is specifically segregated to the early stages of the oligodendrocyte differentiating pathway, and that the receptor is turned down when these cells reach functional maturation (in this respect, see also below).

*In vivo*, GPR17 is also present in both cells of the ependymal layer and in a subset of subependymal precursor cells of the lateral ventricles, one of the classical neurogenic areas that are still active in the adult brain. Again, none of these cells express either GFAP, nestin or DCX, suggesting that they are not “classical” multipotent neurogenic cells or neuronal precursors; moreover, in a similar way to the parenchymal GPR17^+^ precursor cells, they did not incorporate BrdU (at least under the experimental conditions utilized here). These cells may thus represent an additional yet-uncharacterized population of quiescent (or very slowly proliferating) precursors, or may be postmitotic SVZ-derived progenitors progressing along the oligodendroglial differentiation pathway. A similar population of ramified GPR17^+^ cells was also found in the hippocampus, another neurogenic area present in the adult brain.

To verify if GPR17 in oligodendrocytes precursors is indeed functional and could play a role in driving differentiation to mature myelinating cells, primary cortical cultures were treated *in vitro* with UDP-glucose, one of the endogenous agonists of GPR17 [11]. Exposure to this agonist for 72 h resulted in an approximately 3-fold increase of the number of mature MBP^+^ cells. To confirm the specificity of these effects, we demonstrated that the P2Y receptor antagonist cangrelor (that was previously demonstrated to contrast GPR17 activation both *in vitro* and *in vivo* [11], the present study), partially counteracted UDP-glucose induced changes. LTD_4_, which also acts as an agonist at GPR17 ([11]; the present study), induced similar effects, suggesting that activation of GPR17 on oligodendrocyte precursors by its endogenous agonists promotes progression along their differentiation pathway. In treated cultures, the total number of cells was not increased, to suggest that activation of GPR17 is actually inducing the maturation of already existing precursors. No synergistic nor additive effects were noticed when both agonists were combined together. However, since maximal concentrations of both UDP-glucose and LTD_4_ were used in this study, the plateau response may have been already achieved with the single agonist concentration, or the receptor may have undergone agonist-induced desensitization, a typical feature of GPCRs [30]. Future studies employing combinations of different agonist concentrations will clarify this aspect.

In line with these *in vitro* findings, application of a brain injury *in vivo*, such as induction of focal cerebral ischemia, promotes the activation of quiescent GPR17^+^ oligodendrocyte precursors that start proliferating in the parenchyma around the injured area. In the lesioned brain, some of these cells also start expressing more mature myelination markers such as APC, that are never expressed by quiescent GPR17^+^ precursors in the intact telencephalon (see also below).

### Upon brain ischemia, GPR17 is induced in several embryonically different cells that participate to damage evolution and repair

We finally looked at the expression pattern of GPR17 in mice after induction of MCAo. We provide evidence that:

- Upon ischemia induction, there is an early, time-dependent up-regulation of GPR17 in neuronal cells both within and at the borders of the injured area. Numerous neurons over-expressing GPR17 are already evident inside the lesioned area 24 hours after MCAo. Several of these neurons co-localize with inducible HSP70, a marker of cellular stress, injury and death, suggesting a causal link between GPR17 up-regulation and neuronal death. To confirm this potential link, at 48 hours, GPR17^+^ neurons are no longer present inside the lesion suggesting that they have indeed undergone death. To further confirm a causative role of the receptor in induction of cell death inside the ischemic core, the *in vivo* knock down of GPR17 by either a pharmacological or a biotechnological approach markedly reduced the ischemic infarct volume (Figure 7).
- Starting from 48–72 hours after MCAo, at the borders of the lesioned area, we found many GPR17^+^ cells that also strongly stained for IB4, a marker of activated microglia/macrophages. As expected, no IB4^+^ cells are found in the intact cortex, where all microglia was present in its “resting” form (Figure 2K). One week after MCAo, these cells are detected in high amount inside the ischemic lesion (data not shown). This pattern of IB4 positivity indeed reproduces the typical distribution of infiltrating cells in the ischemic brain [31]. It is not possible to establish whether these infiltrating cells are microglia or blood-borne macrophages or both, since under these experimental conditions both types of cells are positive to the same surface and endocellular markers (such as IB4). GPR17 immunoreactivity in these cells is not due to phagocytosis of GPR17 over-expressing neurons by microglia/macrophages, since none of these cells expresses typical neuronal markers such as SNAP25 [32].
- Starting from 72 hours after MCAo, the number of ramified GPR17^+^ oligodendrocyte precursor cells is also significantly increased both around the injured area and in the corpus striatum. These data are in line with previous literature data demonstrating that, in a similar way to neurons, oligodendrocytes are very vulnerable to brain damage *in vivo* and proliferation of oligodendrocyte precursor cells is detected after ischemia around the lesioned area [33], likely as a response to demyelination. Activation of oligodendrocyte precursor cells may contribute to the replenishment of oligodendrocytes and subsequent remyelination in the peri-injury area. In line with this hypothesis, at variance from the intact telencephalon, in ischemic animals, some of the GPR17^+^ parenchymal precursors also expressed typical mature oligodendroglia markers such as MBP (data not shown) and APC, to suggest that, upon damage, quiescent precursors are activated and start progressing along their differentiation pathway. Of course, in future experiments, it will be interesting to verify if, at later times after ischemia, these activated pre-oligodendrocytes start myelinating, thus actually supporting repair of demyelinated lesions.
- In line with previous data demonstrating that ischemic insults activate precursor cells in classic neurogenic areas [25], many BrdU^+^/DCX^+^ cells were found in the SVZ of lesioned mice. However, none of these proliferating cells expresses GPR17, indicating a differential behaviour/regulation of GPR17^+^ precursors in brain parenchyma and in adult neurogenic areas. Based on the demonstration that GPR17 is specifically activated by uracil nucleotides and cysLTs (e.g., UDP-glucose and LTD_4_ [11] and the present paper), and that, as a result of cell damage and death, massive release of nucleotides and cysLTs occurs in the injured area [9] we propose this differential behaviour be due to the fact that only GPR17^+^ precursor cells in the parenchyma have access to and can be activated by their endogenous ligand under these conditions.

### Conclusion

In summary, our study reveals the new cysLT/uracil nucleotide receptor GPR17 as a “sensor” that is activated upon brain injury in several embryonically distinct cell types (neurons, microglia and adult neural precursor cells) that contribute to damage evolution and to the subsequent remodeling and repair. These findings are consistent with the emerging role played by endogenous cysLTs and nucleotides in the injured brain, when these molecules are released from damaged cells, rapidly diffuse in the extracellular environment and act as “danger signals” to alert responses to damage and start repair by ligating specific plasma membrane receptors on target cells. Our unpublished data demonstrate that GPR17 plays a similar role in other models of central nervous system neurodegenerative disease such as spinal cord injury, Alzheimer's disease and multiple sclerosis (manuscripts in preparation); thus, GPR17 may represents a sensor of local injury that is activated upon different kinds of lesions independently of the cause at the basis of damage. Initial up-regulation of GPR17 in neurons is followed by a second wave of receptor induction in microglial/macrophages recruited from distal parenchymal areas and moving towards the lesioned zone.

Our study also reveals GPR17 as an important player in oligodendrocyte differentiation. At variance from other oligodendrogenic determinants, GPR17 is a membrane receptor that can be easily and immediately activated at the site of injury by its endogenous ligands to start remyelination. In addition, pharmacological strategies aiming to activate this receptor on pre-oligodendrocytes via small brain-permeable molecules may also directly contribute to implement intrinsic repair in post-ischemic regeneration as well as in demyelinating neurodegenerative conditions such as multiple sclerosis.

## Materials and Methods

### Cell culture and transfection

Human astrocytoma cells 1321N1 cells were cultured as described [15]. For [^35^S]GTPγS, 10^6^ 1321N1 cells were seeded on 75 cm^2^ flasks and transfected by the calcium phosphate precipitation method [15].

Primary mixed neuronal/glial cultures from cerebral cortex were obtained from E18 Sprague-Dawley rats according to standard procedures [34]. Pregnant animals were euthanized by cervical dislocation under CO_2_ anesthesia, and the fetuses removed and put into ice-cold Hanks balanced salt solution. After dissection of the cortices, cells were dissociated and plated on poly-L-lysine-treated 2.4 cm diameter glass coverslips (3.5×10^5^ cells/coverslip) in Neurobasal (Invitrogen, Gibco) with 2% B27 containing 100 U/ml penicillin, 100 µg/ml streptomycin, 200 mM glutamine, 10 nM glutamate. At 3 days *in vitro* (DIV), half of the culture medium was replaced with fresh medium without glutamate. Double immunofluorescence experiments were performed between 3 to 10 DIV. At 10 DIV, some cultures were treated for 72 h with the following pharmacological agents: 100 µM UDP-glucose (Sigma-Aldrich), in the absence or presence of the P2Y receptor antagonist Cangrelor (10 µM, a gift of the Medicines Company, Parsippany, NJ, USA) or 100 nM LTD_4_ (Cayman Europe, Tallinn, Estonia) or the combined treatment 100 nM LTD_4_+100 µM UDP-glucose, and then fixed for immunostaining.

### RNA isolation, RT-PCR and cloning of mouse GPR17

In the case of mouse tissue and 1321N1 cells, total RNA was extracted using the TRIZOL® Reagent (Invitrogen) according to the manufacturer's instructions. Retrotranscription to cDNA and PCR reactions were carried out as described previously [15]. The following primers were used to detect the expression of mouse GPR17: Fw 5′-GATGAACGGTCTGGAGGCAGCC-3′; Rv 5′-CTCACAGCTCGGATCGGGCAC-3′ (PCR product: 1022 bp, T_A_: 61.4°C). The same primer pair, external to the open reading frame, was used to clone mouse GPR17 (NM_001025381) into a pcDNA3.1 expression vector using the pcDNA3.1/V5-His^©^TOPO® TA Expression Kit (Invitrogen, Milan, Italy). In the case of primary mixed neuronal/glial cultures, one living cell, (or a small group of cells, up to 10), was sucked into a patch pipette by applying a negative pressure, and then processed for RT-PCR, using oligo(dT) for retrotrascription (Invitrogen), as previously described [15]. Choice of oligodendrocyte cells was done at the light microscope, using cell morphology as a guide (see Figure 4 legend). The following primers were used to detect the expression of rat GPR17: Fw 5′-GCTCTTCGCCTGCTTCTACC-3′; Rv 5′-GCGGACGGCTTTATTCTTGA-3′ (PCR product: 610 bp, T_A_: 58.6°C). Twenty µl aliquots of the PCR products were size separated by electrophoresis on a 1,5% agarose gel.

### [^35^S]GTPγS binding assay

Control and transfected 1321N1 cells were homogenized in 5 mM TRIS-HCl, 2 mM EDTA, pH 7.4 and centrifuged at 48000 g for 15 min at 4°C. The resulting plasma membranes were suspended in binding buffer and used in [^35^S]GTPγS binding assay as previously described [11].

### Animals and treatments

Male CD1 mice (Charles River, Calco, Italy) weighing 20–25 g, after intraperitoneally anesthetized with ketamine (0.1 mg/kg) and xylazine (0.02 mg/kg), underwent permanent MCAo, as previously described [35]. Drug treatments were as follows: cangrelor (a single dose of 1.125 µg/animal in 4 µl of saline, a kind gift from The Medicines Company, Parsippany, NJ, USA) was administered intracerebroventricularly (i.c.v.) in the contralateral hemisphere, 20 min after MCAo; anti-sense oligonucleotide 616 and corresponding scrambled oligonucleotide (10 µg/animal, i.c.v., in 4 µl of saline) were administered twice in the contralateral hemisphere, 20 min and 24 h after MCAo. All i.c.v. administrations were performed 1 mm lateral to the middle line, 0.45 mm caudal to bregma, and 2.25 mm depth.

Bromodeoxyuridine (BrdU; 50 mg/kg i.p., Sigma-Aldrich) was given by a single injection in intact mice to evaluate the proliferative activity of GPR17^+^ cells or twice daily for 3 days from 3 hours after MCAo. The animals were then euthanized 1 day, 2, or 3 days after the last injection. This study on animals has been approved by the Council of the Department of Pharmacological Sciences of the University of Milan, Italy, which is legally entitled for the use of animals for scientific proposes (D.M. of Italian Ministry of Health, Authorization # 124/2003 of 13/10/2003 and #143/2004 of 13/12/2004), which is, in turn, based on the D.L. of 27 January 1992 nr. 116. Implementation of European Union Directive nr. 86/609/CEE regarding the Protection of animals used for experimental and other scientific purposes.

### MRI analysis of ischemic damage development

Brain infarct size was visualized by MRI measurements taken 2, 24 and 48 h after MCAo using a 4.7 T, vertical super wide bore magnet of a Burker Avance II spectrometer with micro imaging accessory. Animals were anesthetized with 1.5% isofluorane in 70% N_2_ 30% O_2_, fixed on the holder and placed into the 3.8 cm diameter birdcage coil. A 3-orthogonal-plane, gradient echo scout acted as a geometric reference for locating the olfactory bulb; then, caudally, 12 contiguous coronal DW slices were acquired. The field of view was 2.56×1.6 cm^2^, the in plane resolution was 200×200 µm^2^ and the slice thickness was 800 µm in all the images. Spin echo DW images were acquired with 40 ms echo time and 1.5 s repetition time. Diffusion weighting was obtained by two 10 ms long and 25 ms spaced gradients resulting in a b value of about 900 s/mm^2^. Trace of diffusion tensor map computation, ischemic volume determination and progression of the ischemic damage over time were as described [36].

### Western Blot analysis

Total protein extracts were obtained by tissue homogenization in 4 mM HEPES buffer, pH 7.3, containing 250 mM sucrose and protease inhibitors. The homogenate was centrifuged at 800 g for 10 min at 4°C, and then the supernatant was centrifuged at 200000 g for 1 h at 4°C to generate membrane and cytosolic fractions. The protein concentration was estimated by the Bradford procedure (Bio-Rad Laboratories). Equal amounts of protein from plasma-membranes and cytosolic fractions (30 µg) were loaded on 10% SDS-PAGE and blotted on nitrocellulose paper (Invitrogen). Nitrocellulose membranes were then incubated with rabbit anti-GPR17 polyclonal antibody (1∶1000) for 2 h at RT. Specific signals were detected using the ECL immunoblotting detection system (Amersham Life Science).

### Immunohistochemistry, confocal and fluorescence microscopy

GPR17 expression pattern was evaluated and confirmed on paraffin-embedded, frozen and floating brain sections. For paraffin embedding, the removed brains were fixed in Carnoy reagent, embedded in Paraplast (Sigma-Aldrich), and 8 µm coronal sections were processed for immunohistochemistry as previously described [11]. For frozen and floating sections, the animals have been perfused with with 4% paraformaldehyde in 0.01 M PBS and then incubated for 24 hours in a solution of 30% sucrose, embedded in OCT (Cell Path) and then frozen at −80°C. After perfusion and brain cryoprotection, 14 µm frozen sections or 30 µm floating sections were cut with a microtome or a vibratome, respectively, and treated as in [16]. Double labeling of brain sections was performed using the rabbit anti-GPR17 polyclonal antibody (1∶100) generated as previously described [11] in combination with the selected primary antibody, in PBS 0.01 M, 0.1% Triton X-100. For co-localization studies, the following mouse monoclonal antibodies were used: anti-SMI311 (1∶300, Sternberger Monoclonal Inc.), anti-NeuN (1∶300, Chemicon), anti-GFAP (1∶600, Cell Signalling), anti-S100β (1∶1000, Sigma-Aldrich), anti-CNPase (1∶100, Chemicon), anti-MAG (1∶500, Chemicon), anti-nestin (1∶200, Chemicon), anti-HSP70 (1∶3000, Sigma-Aldrich), anti-APC CC-1 clone (1∶50, Calbiochem). In addition, the rat monoclonal anti-myelin basic protein (MBP; 1∶200, Chemicon) and anti bromodeoxyuridine (1∶150, Abcam) were used. Double labelings with the rabbit anti-Olig2 (1∶800, Chemicon), anti-NG2 (1∶200, Chemicon), doublecortin (DCX; 1∶100, Cell Signalling), anti-Iba1 (1∶1000, Wako Chemicals, Neuss, Germany), and with isolectin B4 (IB4) HRP-conjugated (1∶100, Sigma-Aldrich) were also performed. When co-staining with primary antibodies developed in the same species, GPR17 was detected with the high sensitivity tyramide signal amplification kit (Perkin Elmer). Double labeling with anti-NG2 was performed using detergent free buffers.

For detection, goat anti-rabbit AlexaFluor 555 (1∶500), goat anti-rat AlexaFluor 555 (1∶500), goat anti-mouse AlexaFluor 488 (1∶600, all from Molecular Probes) or goat FITC-conjugated anti-HRP (1∶100, Jackson Immunoresearch) were used. In selected experiments an additional step with the UV fluorescent dye Hoechst 33258 (1∶50000, Molecular Probes) for the nuclei labeling was performed.

### Immunocytochemistry

Immunofluorescence analysis of cortical cultures was performed as previously described [37]. Double immunostaining was performed incubating fixed cultures with anti-GPR17 polyclonal antibody (1∶150, 2.5 h at room temperature) [11] and one of the following primary antibodies: mouse anti-β-Tubulin-III (β-TubIII; 1∶100), mouse anti-nestin (1∶100), mouse anti-CNPase (1∶100), rat anti-MBP (1∶200), mouse anti-O4 (1∶100, all from Chemicon), mouse anti-NG2 (1∶200, Abcam), mouse anti-MAP2 (1∶500, Immunological Sciences), and mouse anti-GFAP (1∶500, Cell Signalling). Cells were then incubated for 1 h at RT with the secondary goat anti-rabbit and goat anti-mouse antibodies, conjugated to AlexaFluor 488 or AlexaFluor 555 (1∶600, Molecular Probes). Coverslips were mounted with Fluorescent mounting medium (Dako), and analyzed by using an inverted fluorescence microscope (200 M; Zeiss) equipped with a CCD camera (AxioCam HRm; Zeiss), connected to a PC equipped with the Axiovision software (Zeiss).

### Propidium Iodide (PI) staining

To evaluate the viability of *in vitro* cell cultures, after pharmacological treatments, cultures were loaded with 20 µM PI for 10 min. Under these conditions, PI can only enter cells with damaged permeabilized membrane and cannot label nuclei of intact viable cells. Cultures were then fixed with 4% paraformaldehyde plus 4% sucrose, and loaded with the nuclear dye Hoechst 33258. Nuclei were analyzed and counted using a fluorescence microscope (Zeiss). Since Hoechst 33258 gives a blue colour to nuclei under UV light, cells that remained alive during the pharmacological treatments showed blue nuclei with rounded appearance; conversely, cells that underwent cell death showed a red colour due to the incorporation of both dyes and were classified as dead. Data are presented as percentage of propidium iodide-positive nuclei over Hoechst 33258-labeled nuclei.

### Data analysis

For [^35^S]GTPγS binding data analysis and graphic presentation, a non-linear multipurpose curve-fitting program Graph-Pad Prism was used and the EC_50_ values were derived. Data are reported as mean±SEM of three different experiments (performed in duplicate).

For MRI studies, data on progression of ischemic damage over time were evaluated by ANOVA for repeated measures. For each animal, the ischemic area at 2 h after ischemia induction was set to 100%, and the extension of the ischemic area at all other time points was proportionally calculated, thus providing an internal control of ischemia development. Then, variations of ischemic volumes between animals and groups were compared. Six animals/group were used. Data are expressed as mean values±s.em. P values ≤0.05 were considered statistically significant.

Quantifications of immunoreactive cells were performed by fluorescence microscopy blindly in coronal sections of three intact and three lesioned hemispheres. Cells were counted using a 20× objective on an inverted fluorescence microscope (200 M; Zeiss). Depending on the experiment and on the parameters to be estimated, at least 700 cells/condition were counted.

### Ethical declaration

This study on animals has been approved by the Council of the Department of Pharmacological Sciences of the University of Milan, Italy, which is legally entitled for the use of animals for scientific proposes (D.M. of Italian Ministry of Health, Authorization # 124/2003 of 13/10/2003 and #143/2004 of 13/12/2004), which is, in turn, based on the D.L. of 27 January 1992 nr. 116. Implementation of European Union Directive nr. 86/609/CEE regarding the Protection of animals used for experimental and other scientific purposes.

## Supporting Information

Figure S1 Endogenous and heterologous expression of murine GPR17 (A) RT-PCR analysis of GPR17 in various mouse tissues. Specific transcripts are found in brain and, to a lower extent, in kidney, heart and skeletal muscle. No detectable expression is found in liver and lung. Amplification of β-actin is shown as an internal reaction control. (B) 1321N1 human astrocytoma cells were heterologously transfected with either pcDNA3.1 empty vector or pcDNA3.1-Gpr17m, as indicated. RT-PCR analysis shows the presence of the receptor transcript at the expected length of 1022 bp in the lane corresponding to the Gpr17 transfected cells. No amplification products are detected in the absence of retro-transcription (indicated as -) or in cells transfected with the empty vector. (C) Fluorescence immunocytochemistry in 1321N1 cells shows that no expression of GPR17 can be detected in cells transfected with pcDNA3.1 empty vector incubated with the anti-GPR17 antibody (left panel), whereas a specific signal (green fluorescence) is present in cells transfected with pcDNA3.1-Gpr17m (center panel). To confirm the specificity of the signal, preincubation of the antibody with its neutralizing peptide abolishes GPR17 positivity in these cells (right panel). Scale bar: 30 µm.
(0.49 MB TIF)

Figure S2 Association of GPR17^+^ cells with myelinating fibers. Double staining fluorescence microscopy image of GPR17^+^ ramified precursor cells (green fluorescence) and MBP^+^ myelinating tracts (red fluorescence) in the intact c. striatum. Immature GPR17^+^ precursors cells do not co-express MBP but are often found physically associated to or inside MBP^+^ myelinating fibers. These cells may represent a source of pre-oligodendrocytes able to turn into myelinating cells when necessary; alternatively, they could play a role in the local trophic control of myelination. Scale bar: 50 µm.
(0.54 MB TIF)

Figure S3 Potential Olig2 binding sites upstream of the GPR17 coding sequence. Sequence alignments of 8 Kb upstream to the coding sequence of the human, rat and mouse GPR17 gene have been performed using BLAST program from NCBI (http://blast.ncbi.nlm.nih.gov). Identification of responsive sites for Neurogenin and NeuroD family of transcription factors (such as Atoh1, Neurod1, Neurod2, Neurod4, Neurod6, Neurog1, Neurog2, Neurog3, TGF12 and TGF3 and Olig2) in the upstream sequences of GPR17 (human, rat and mouse) was performed by using MatInspector Release Professional 7.7.3 software from Genomatix. GPR17 upstream sequences globally display a low homology rate in the three species, except in six regions which are highly homologous and conserved among phylogenesis. Here we show only the two of them (E-Box 1 and E-Box 2) in which the E-Box element, potentially able to bind Olig2, is identical in human, mouse and rat.
(0.36 MB TIF)
