# Supplementary figures and images for: The Recently Identified P2Y-Like Receptor GPR17 Is a Sensor of Brain Damage and a New Target for Brain Repair
Source: PLoS One. 2008 Oct 31;3(10):e3579. doi: 10.1371/journal.pone.0003579 (PMC2570486; doi:10.1371/journal.pone.0003579)

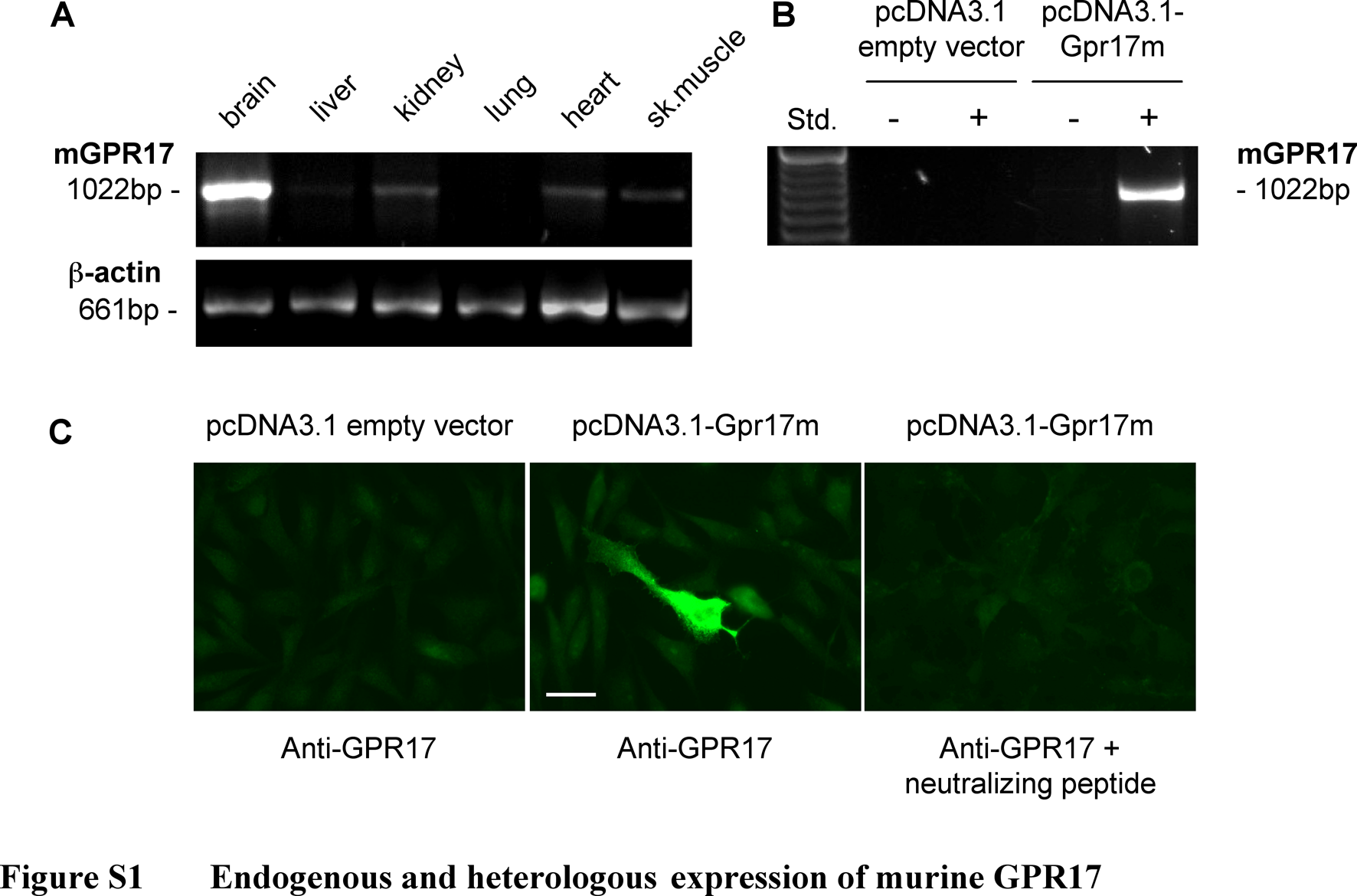

Supplement: Figure S1 — Endogenous and heterologous expression of murine GPR17 (A) RT-PCR analysis of GPR17 in various mouse tissues. Specific transcripts are found in brain and, to a lower extent, in kidney, heart and skeletal muscle. No detectable expression is found in liver and lung. Amplification of β-actin is shown as an internal reaction control. (B) 1321N1 human astrocytoma cells were heterologously transfected with either pcDNA3.1 empty vector or pcDNA3.1-Gpr17m, as indicated. RT-PCR analysis shows the presence of the receptor transcript at the expected length of 1022 bp in the lane corresponding to the Gpr17 transfected cells. No amplification products are detected in the absence of retro-transcription (indicated as -) or in cells transfected with the empty vector. (C) Fluorescence immunocytochemistry in 1321N1 cells shows that no expression of GPR17 can be detected in cells transfected with pcDNA3.1 empty vector incubated with the anti-GPR17 antibody (left panel), whereas a specific signal (green fluorescence) is present in cells transfected with pcDNA3.1-Gpr17m (center panel). To confirm the specificity of the signal, preincubation of the antibody with its neutralizing peptide abolishes GPR17 positivity in these cells (right panel). Scale bar: 30 µm. (0.49 MB TIF) [file pone.0003579.s001.tif]

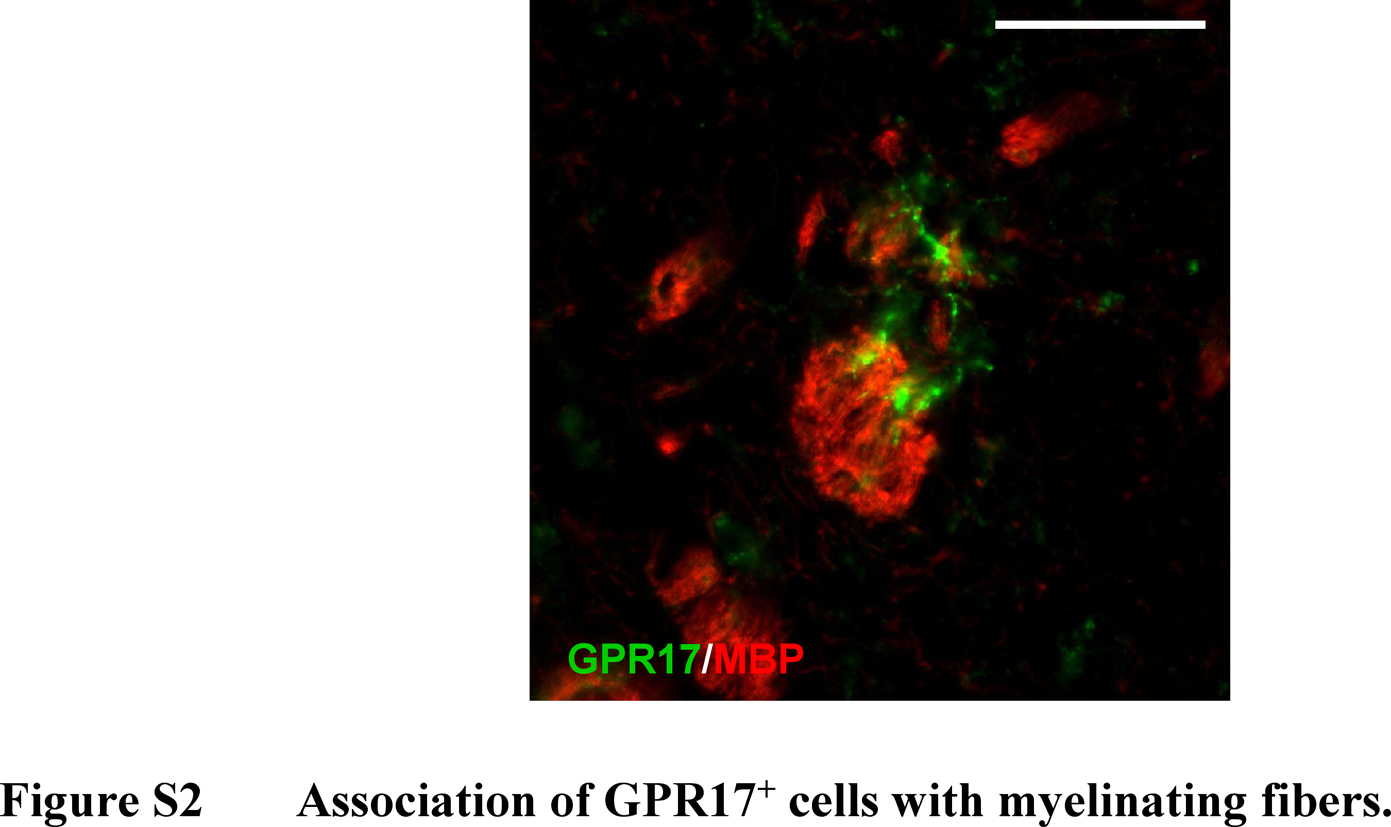

Supplement: Figure S2 — Association of GPR17+ cells with myelinating fibers. Double staining fluorescence microscopy image of GPR17+ ramified precursor cells (green fluorescence) and MBP+ myelinating tracts (red fluorescence) in the intact c. striatum. Immature GPR17+ precursors cells do not co-express MBP but are often found physically associated to or inside MBP+ myelinating fibers. These cells may represent a source of pre-oligodendrocytes able to turn into myelinating cells when necessary; alternatively, they could play a role in the local trophic control of myelination. Scale bar: 50 µm. (0.54 MB TIF) [file pone.0003579.s002.tif]

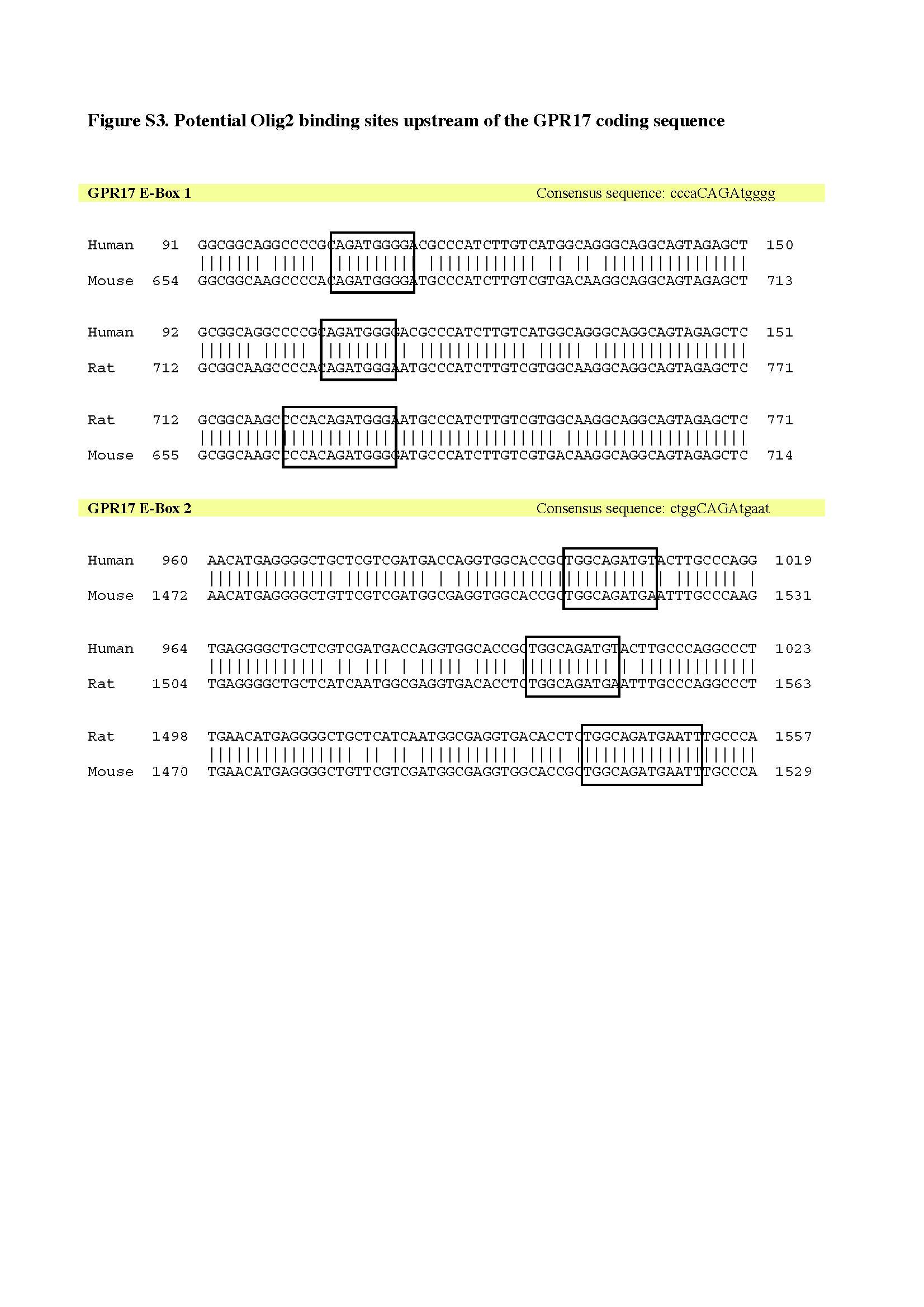

Supplement: Figure S3 — Potential Olig2 binding sites upstream of the GPR17 coding sequence. Sequence alignments of 8 Kb upstream to the coding sequence of the human, rat and mouse GPR17 gene have been performed using BLAST program from NCBI (http://blast.ncbi.nlm.nih.gov). Identification of responsive sites for Neurogenin and NeuroD family of transcription factors (such as Atoh1, Neurod1, Neurod2, Neurod4, Neurod6, Neurog1, Neurog2, Neurog3, TGF12 and TGF3 and Olig2) in the upstream sequences of GPR17 (human, rat and mouse) was performed by using MatInspector Release Professional 7.7.3 software from Genomatix. GPR17 upstream sequences globally display a low homology rate in the three species, except in six regions which are highly homologous and conserved among phylogenesis. Here we show only the two of them (E-Box 1 and E-Box 2) in which the E-Box element, potentially able to bind Olig2, is identical in human, mouse and rat. (0.36 MB TIF) [file pone.0003579.s003.tif]
